# Safety and Efficacy of Peroral Endoscopic Shorter Myotomy versus Longer Myotomy for Patients with Achalasia: A Systematic Review and Meta-analysis

**DOI:** 10.1155/2022/6770864

**Published:** 2022-03-30

**Authors:** Han Zhang, Xinyi Zeng, Shu Huang, Huifang Xia, Lei Shi, Jiao Jiang, Wensen Ren, Yan Peng, Muhan Lü, Xiaowei Tang

**Affiliations:** ^1^Department of Gastroenterology, The Affiliated Hospital of Southwest Medical University, Luzhou, China; ^2^Department of Gastroenterology, The People's Hospital of Lianshui, Huaian, China

## Abstract

**Background and Aims:**

The adequate myotomy length during peroral endoscopic myotomy (POEM) is still controversial. We performed this systematic review and meta-analysis to determine the efficacy and safety of the modified POEM with shorter myotomy (SM) and compare the outcomes between SM and longer myotomy (LM) in achalasia patients.

**Methods:**

A comprehensive literature search was conducted in PubMed, EMBASE, Cochrane Library, and Web of Science databases from inception to May 28, 2021. The primary outcome was clinical success rate and incidence of reflux-relative adverse events (AEs). Fixed- or random-effect models were adopted for the analysis according to the heterogeneity.

**Results:**

Five studies involving 225 patients in SM group and 222 patients in LM group were included. The overall clinical success of SM was 96.6% (95% confidence interval (CI) 92.7 to 98.4%). SM showed noninferior response as compared to LM (risk ratio (RR) 1.02, 95% CI 0.98 to 1.06, *P* = 0.41, *I*^2^ = 0%). Based on the abnormal acid reflux by pH monitoring, its incidence was significantly lower in the SM group than that in the LM group (RR 0.58, 95% CI 0.36 to 0.94, *P* = 0.03, *I*^2^ = 0%). With respect to procedure-related parameters, the total procedure time of SM was significantly shorter than that of LM (mean difference (MD) -16.30, 95% CI -23.10 to -9.49, *P* < 0.001, *I*^2^ = 68%).

**Conclusions:**

SM and LM are comparable in providing treatment efficacy for achalasia patients, whereas less operation time and lower incidence of post-POEM abnormal esophageal acid exposure are observed in SM.

## 1. Introduction

Achalasia is a relatively rare motility disorder of the esophagus characterized by insufficient lower esophageal sphincter (LES) relaxation and abnormal peristalsis, resulting in progressive dysphagia to liquids and solids, regurgitation of undigested food, noncardiac chest pain, and different degrees of weight loss [1]. Achalasia is incurable because the underlying etiology remains unknown. It has been reported that the primary cause of achalasia may be the selective loss of inhibitory neurons in the myenteric plexus of the distal esophagus and LES [2]. As a result, all available therapeutic options of achalasia currently are palliative and aimed to lower LES pressure to improve esophageal emptying, including medical managements such as oral pharmacological therapy, endoscopic botulinum toxin injection, endoscopic pneumatic dilatation, laparoscopic Heller myotomy, and peroral endoscopic myotomy (POEM) [3].

POEM was first performed by Inoue et al. in 17 patients with achalasia nearly a decade ago [4]. For the first seven patients in their study, a relatively shorter myotomy (SM) (mean 4.9 cm) was used, while for the last ten patients, a longer myotomy (LM) (mean 10.4 cm) was used, and it was found that the latter group experienced better symptom improvement [4]. Since then, thousands of POEM procedures with LM have been adopted worldwide for patients with achalasia, and a large number of clinical studies and meta-analyses have reported its excellent efficacy and safety with a reported mean myotomy length range from 8.2 to 14.4 cm [5–7]. However, achalasia is a LES dysfunction disorder and the length of LES is reported just 3.6 cm (range from 3.3 to 4.3 cm) in achalasia patients [8]. Hence, modified POEM with SM might be able to provide the same benefits on patients with achalasia as the LM. Another key point is that lowering LES pressure not only leads to symptom relief but also increases lower esophageal acid exposure, with the high risk of post-POEM gastroesophageal reflux disease (GERD) [9]. Meanwhile, a previous study has demonstrated that gastric myotomy > 2.5 cm resulted in increased rates of moderate esophagitis [10].

Presently, the optimal myotomy length remains unknown due to the lack of evidence, but a few papers have reported the promising clinical outcomes of the modified POEM with SM for achalasia patients [8, 11–14]. To provide more practical recommendations for endoscopists, we performed this systematic review and meta-analysis to determine the efficacy and safety of the modified POEM with SM (myotomy length ≤ 7 cm) [4] and compare the clinical success rate and incidence of reflux-relative adverse events (AEs) between SM and LM (myotomy length > 7 cm) in achalasia patients [4].

## 2. Methods

The Preferred Reporting Items for Systematic Reviews and Meta-Analyses (PRISMA) 2020 statement [15] was followed in this systematic review and meta-analysis [15]. We stated that the protocol of this review was not registered. As it was studied based on the published summary data, written consent from patients and ethical approval from an institutional review board were not required.

### 2.1. Eligibility Criteria

Prespecified inclusion criteria were as follows: (1) population: adult individuals (age greater than 18 years) who were diagnosed with achalasia based on symptoms, endoscopy, barium swallow, and high-resolution manometry (HRM) [1]; (2) intervention: the modified POEM with SM (total myotomy length ≤ 7 cm with about 2 cm incision at the gastric side) [4]; (3) comparison: no comparison or conventional POEM with LM (total myotomy length > 7 cm) [4]; (4) outcomes: provided data on primary outcomes, including clinical success (Eckardt score ≤ 3) and/or reflux-related adverse events [16]; and (5) study type: all controlled, uncontrolled, prospective, and retrospective articles.

Prespecified exclusion criteria were as follows: (1) meta-analysis, reviews, case reports, case series, experimental studies in animal models, conference abstracts, editorials, letters to the editor, and expert comments; (2) studies with incomplete data or ongoing trials without reported clinical outcomes; and (3) duplicate studies with overlapped patients except for the most recent publication with the largest population.

### 2.2. Information Sources and Search Strategy

Two authors (Shu Huang and Huifang Xia) independently conducted a comprehensive literature search in PubMed, EMBASE, Cochrane Library, and Web of Science [v.5.35] databases from inception to May 28, 2021, without language restriction. The following search keywords were adopted: “POEM” and “achalasia”. Disagreements were resolved by consensus. The detailed search strategies and identified items in each database are presented in Supplementary Table 1. Additionally, we examined the references of the screened records and searched significant articles manually to identify additional studies.

### 2.3. Selection Process

After using an automated tool to remove duplicates, the authors (Shi Lei and Xia Huifang) independently screened all titles and abstracts with retained records found in a literature search. Irrelevant studies were excluded. The steps so far have been done in the EndNote software. The authors then independently reviewed the full text of the remaining records and identified eligible studies according to our inclusion and exclusion criteria items. Mismatched studies were excluded. Differences of opinion on the choice of research at the level of title/abstract or full text should be resolved through consensus and discussion with the third author (Zhang Han). To summarize the study selection process, we used a modified PRISMA flowchart [15].

### 2.4. Data Collection Process and Data Items

Two authors (Jiao Jiang and Wensen Ren) independently used a standardized spreadsheet that had been developed in advance to extract the data from the eligible studies. Disagreements were resolved by consensus and discussion with a third author (Han Zhang). When an included study failed to supply us with relevant information, we contacted the authors of the paper by email to seek extra details.

The primary outcomes were as follows: (1) the overall clinical success rate in SM group and the difference of clinical success rate between SM and LM groups. We restricted the Eckardt score as a measure of clinical success in our analysis. The Eckardt score consists of four symptoms (dysphagia, regurgitation, chest pain, and weight loss) that are graded according to severity, and the clinical success is defined as a score ≤ 3 [16]. (2) Postoperative reflux-related events including symptomatic reflux, reflux esophagitis on endoscopy, and abnormal acid reflux based on pH monitoring. The secondary outcomes were as follows: (1) the difference of the perioperative outcomes including total procedure time and hospital stay between the SM and LM groups; (2) the difference between pre- and postoperative outcomes including Eckardt score, lower esophageal sphincter pressure (LESP), integrated relax pressure (IRP), and diameter of barium column (DBC) in the SM group; (3) the difference of postoperative outcomes between SM and LM including Eckardt score, LESP, and IRP; and (4) the overall technical success and the number of various types of perioperative adverse events (AEs) in the SM group.

The following data were extracted from each article: (1) study characteristics: first author, year of publication, study design, study period, study location, and follow-up duration; (2) patients' demographics in both the SM and LM groups: sample size, age, sex, symptoms duration, Chicago classification, and previous treatments; (3) POEM procedure details in both the SM and LM groups: total procedure time, myotomy direction, tunnel length, myotomy length, and hospital stay; and (4) reported primary and secondary outcomes. The data that support the results of this study are available from Dr. Han Zhang (443191590@qq.com) upon reasonable request.

### 2.5. Study Risk of Bias Assessment

Two authors (Jiao Jiang and Wensen Ren) independently identified and evaluated the risk of bias of the included studies. The methodological quality of the observational studies was assessed using the Newcastle-Ottawa scale (NOS) [17], which assesses selection (4 items), comparability (2 items), and outcomes (3 items). A study can be awarded a maximum of one star for each item within the selection and outcome categories while a maximum of two stars can be given for comparability. Generally, studies with no less than six stars were considered of high quality. The methodological quality of the randomized controlled trials (RCTs) was assessed using the Cochrane Collaboration's tool [18], which covers six domains of bias: selection bias, performance bias, detection bias, attrition bias, reporting bias, and other bias. A judgment of high, low, or unclear risk of material bias was given to each item. Any discrepancies were resolved by consensus and discussion with a third author (Han Zhang) during the quality assessment.

### 2.6. Reporting Bias Assessment

To detect outcome reporting bias, we examined the trial protocols to see if the specified outcomes were reported in the corresponding trial publications. When trial protocols were not available, we compared the outcomes reported in the methods and results sections of the trial publications. We did not statistically perform funnel plot asymmetry test and Egger's test to assess publication bias because there were only five papers included.

### 2.7. Certainty Assessment

Two reviewers (Yan Peng and Muhan Lü) independently assessed the quality of the evidence for results from the meta-analysis using the Grading of Recommendations Assessment, Development and Evaluation Working Group (GRADE) system [19]. The system classifies the overall quality of evidence as high, moderate, low, or very low four levels. Firstly, the rating of the estimate from observational studies begins with low-quality evidence, while the rating of the estimate from randomized controlled trials begins with high-quality evidence. Then, it can decrease based on the five considerations that include the study limitations, inconsistency of results, indirectness of evidence, imprecision, and reporting bias, whereas it can increase based on large effect, plausible confounding, and dose response. During this process, any disagreements were resolved by consensus and discussion with a third author (Han Zhang).

### 2.8. Statistical Analysis

We performed a meta-analysis if data were available for more than one study. For meta-analyses of continuous variables (total procedure time, hospital stay, Eckardt score, LESP, IRP, and DBC), the mean differences (MD) between pre- and post-POEM data or between SM and LM data were calculated with 95% confidence intervals (CIs). All continuous data reported as mean/median (range) values were converted to mean ± SD before analysis according to the method of Hozo et al. [20]. For meta-analyses of dichotomous variables (technical success, clinical success, and reflux-related AEs), the pooled event rate in SM and the risk ratio (RR) between SM and LM data were calculated with 95% CIs. Heterogeneity among studies was qualitatively and quantitatively assessed using two methods: the *χ*^2^ test (*P* < 0.10 indicated the presence of heterogeneity) and *I*^2^ statistic. *I*^2^ values of 0-50%, 51-74%, and 75% or more were considered to indicate a low, moderate, and high degree of heterogeneity, respectively. In the presence of substantial heterogeneity (*I*^2^ > 50%), a random-effect model was used as a pooling method; otherwise, a fixed-effect model was adopted. We were unable to perform subgroup analyses of characteristics such as symptom duration, achalasia subtype, and previous treatments owing to insufficient data. Sensitivity analyses were also conducted by using the leave-one-out method to test the influence of each individual study on pooled estimates. All *P* values were 2-tailed, and *P* values < 0.05 were considered statistically significant in all tests except for the *χ*^2^ test. All statistical procedures were conducted using the statistical software Review Manager 5.3 with the exception of the pooled event rate, which was performed in Comprehensive Meta-Analysis software.

## 3. Results

### 3.1. Study Selection

**Figure 1:**
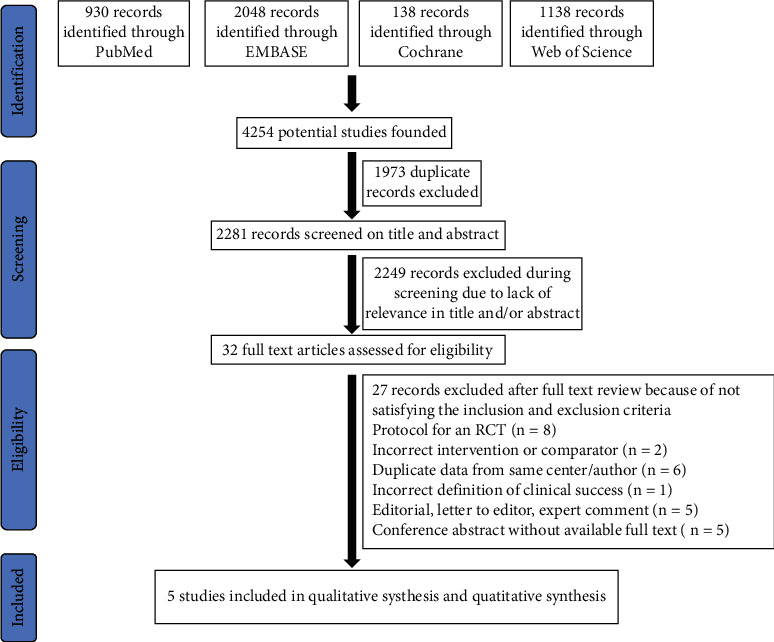
Adapted Preferred Reporting Items for Systematic Reviews and Meta-Analyses flowchart.

The initial literature databases search yielded 4254 potential related records, of which 930 on PubMed, 2048 on EMBASE, 138 on Cochrane Library, and 1138 on Web of Science. The records were transferred to the EndNote for screening, and 1973 duplicates were removed using automation tools. Then, out of the 2281 remaining studies, 2249 irrelevant studies were eliminated after assessing their title and abstract. Finally, out of the 32 remaining studies, 27 studies were excluded after the examination of their full text based on the inclusion and exclusion criteria. No additional study was retrieved from the references of the screened records. The reasons and references for the excluded studies after full text review were available in Supplementary Table 2. Five studies [8, 11–14] involving 225 patients in SM and 222 patients in LM were included in final qualitative analysis and quantitative synthesis. The adapted flow diagram of the study selection is presented in Figure 1.

### 3.2. Study Characteristics

The main characteristics of the included studies and patients are described in Table 1. Five studies [8, 11–14] with a total of 447 patients were included, of which four studies [11–14] compared the clinical outcomes between SM and LM (225 patients in the SM group and 222 patients in the LM group). Two RCTs [13, 14], one prospective cohort study [8], and two retrospective cohort studies [11, 12] were analyzed with a short-term follow-up. All studies were performed in the East Asia, including 4 in China and 1 in India. The period of patient enrollment was between 2011 and 2019. The sample size varied from 34 to 63 in the SM group and from 37 to 74 in the LM group. The mean age ranged from 36 to 49.3 years in the SM group and from 37.7 to 45.9 years in the LM group. The male proportion ranged from 37% to 53% in the SM group and from 48% to 65% in the LM group. The symptom duration varied widely from 0.7 to 9.4 years in both the SM and LM groups. Based on Chicago classification, there were 66 type I, 156 type II, and 3 type III achalasia in the SM group and 48 type I, 172 type II, and 2 type III achalasia in the LM group. The detailed characteristics of the POEM procedures are presented in Table 2. The mean total procedure time ranged from 31.2 to 52 minutes in the SM group and from 45.6 to 72.43 in the LM group. The mean length of hospital stay ranged from 2.82 to 9.9 days in the SM group and from 2.81 to 9.3 days in the LM group. The detailed outcomes reported in the included studies are summarized in Table 3. All POEM procedures were performed successfully, and no surgery intervention was required. The reported clinical success rate ranged from 94.4% to 100% in the SM group and from 91.9% to 98% in the LM group.

### 3.3. Risk of Bias in Studies

NOS was used to assess the risk of bias for 3 cohort studies. All cohort studies were given a score of 6-7 stars, representing the high quality of studies. Cochrane Collaboration's tool was used to assess the risk of bias for 2 RCTs, and only other bias was unclear in both two studies, representing that all RCTs were of high quality. The results of NOS and Cochrane Collaboration's tool quality assessment are summarized in Supplementary Table 3 and Supplementary Table 4, respectively.

### 3.4. Overall Clinical Success and Technical Success in the SM Group

**Figure 2:**
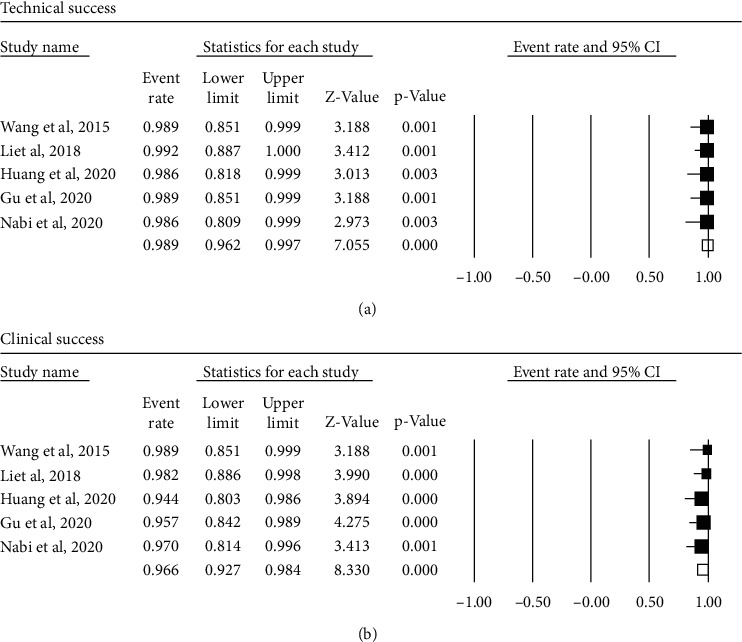
Forest plot presenting the pool event rate for technical success (a) and clinical success (b) of the modified peroral endoscopic myotomy with shorter myotomy in achalasia.

Five studies that included a total of 225 patients were available to estimate the overall clinical success rate of SM. We used a fixed-effect model due to insignificant heterogeneity (*I*^2^ = 0%, *P* = 0.775), and the pooled clinical success rate of POEM with SM for achalasia patients was estimated at 96.6% (95% CI 92.7 to 98.4%) (Figure 2(b)). For technical success, the estimated pooled event rate was 98.9% (95% CI 96.2 to 99.7%; *I*^2^ = 0%, *P* = 0.998, Figure 2(a)).

### 3.5. Pre-POEM versus Post-POEM in the SM Group

**Figure 3:**
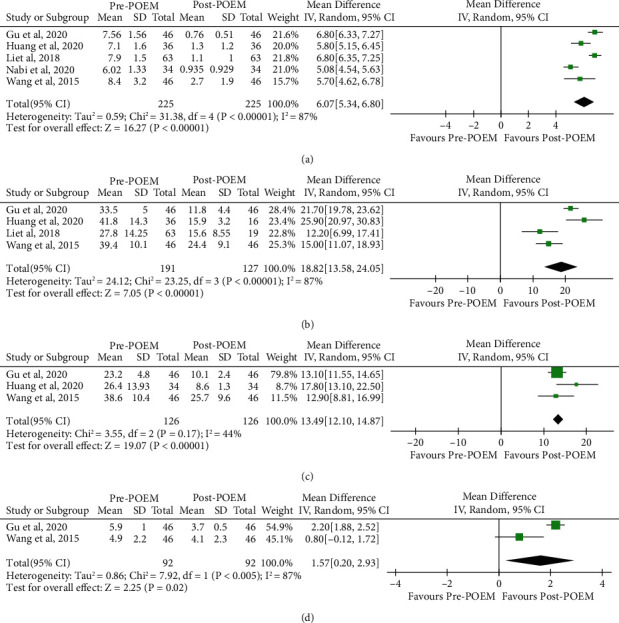
Forest plot presenting the mean difference of Eckardt score (a), lower esophageal sphincter pressure (b), integrated relax pressure (c), and diameter of barium column (d) between before and after peroral endoscopic myotomy with shorter myotomy in achalasia.

Five studies involving 225 patients in the SM group compared pre-POEM with post-POEM outcomes. As the heterogeneity among studies was significant in Eckardt score, LESP, and DBC (*I*^2^ = 87%, *P* < 0.001; *I*^2^ = 87%, *P* < 0.001; *I*^2^ = 0%, *P* = 0.005, respectively), we used random-effect model for the analysis. While the heterogeneity among studies was low in IRP (*I*^2^ = 44%, *P* = 0.17), we used fixed-effect model for the analysis. In terms of the Eckardt score, achalasia patients treated with SM showed significant response as compared to pre-POEM (4 studies, *n* = 225 in the pre-POEM arm and *n* = 225 in the post-POEM arm, MD 6.07, 95% CI 5.34 to 6.20, Figure 3(a)). Based on the LESP, achalasia patients treated with SM showed significant improvement as compared to pre-POEM (4 studies, *n* = 191 in the pre-POEM arm and *n* = 127 in the post-POEM arm, MD 18.82, 95% CI 13.58 to 24.05, Figure 3(b)). With respect to the IRP, achalasia patients treated with SM showed significant response as compared to pre-POEM (3 studies, *n* = 126 in the pre-POEM arm and *n* = 126 in the post-POEM arm, MD 13.49, 95% CI 12.10 to 14.87, Figure 3(c)). For the DBC, achalasia patients treated with SM showed significant improvement as compared to pre-POEM (2 studies, *n* = 92 in the pre-POEM arm and *n* = 92 in the post-POEM arm, MD 1.57, 95% CI 0.20 to 2.93, Figure 3(d)).

### 3.6. SM versus LM in Terms of Efficacy

**Figure 4:**
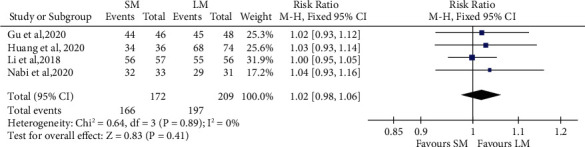
Forest plot presenting the risk ratio of clinical success between shorter myotomy and longer myotomy of peroral endoscopic myotomy in achalasia.

**Figure 5:**
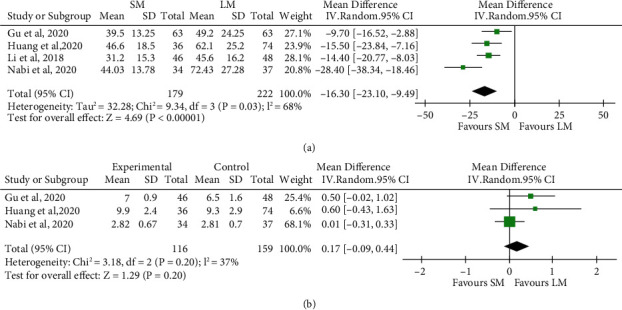
Forest plot presenting the mean difference of total procedure time (a) and length of hospital stay (b) between shorter myotomy and longer myotomy of peroral endoscopic myotomy in achalasia.

**Figure 6:**
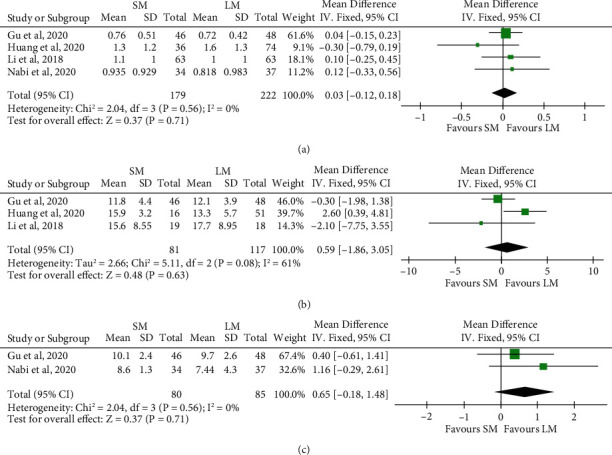
Forest plot presenting the mean difference of post-POEM Eckardt score (a), lower esophageal sphincter pressure (b), and integrated relax pressure (c) between shorter myotomy and longer myotomy of peroral endoscopic myotomy in achalasia.

Four studies compared clinical outcomes of SM with LM involving 179 patients in SM and 222 patients in LM. In terms of clinical success, as the heterogeneity among studies was low (*I*^2^ = 0%, *P* = 0.89), we used fixed-effect model for the analysis. Achalasia patients treated with SM showed noninferior response as compared to LM (4 studies, *n* = 172 in the SM arm and *n* = 209 in the LM arm, RR 1.02, 95% CI 0.98 to 1.06, *P* = 0.41, Figure 4). With respect to the procedure-related parameters, as the heterogeneity among studies with regard to the total procedure time was significant (*I*^2^ = 68%, *P* = 0.03), we used random-effect model for the analysis. Meanwhile, as the heterogeneity among studies with regard to the length of hospital stay was low (*I*^2^ = 37%, *P* = 0.20), we used fixed-effect model for the analysis. The total procedure time of SM was significantly shorter than that of LM (4 studies, *n* = 179 in the SM arm and *n* = 222 in the LM arm, MD -16.30, 95% CI -23.10 to -9.49, *P* < 0.001, Figure 5(a)). However, the length of hospital stay did not differ significantly between the groups (3 studies, *n* = 116 in the SM arm and *n* = 159 in the LM arm, MD 0.17, 95% CI -0.09 to 0.44, *P* = 0.20, Figure 5(b)). For Eckardt score, LESP, and IRP, the LM seemed to show more improvement compared with SM; however, the difference between the two groups were not found to be statistically significant (Figure 6).

### 3.7. SM versus LM in Terms of Reflux-Related Events

**Figure 7:**
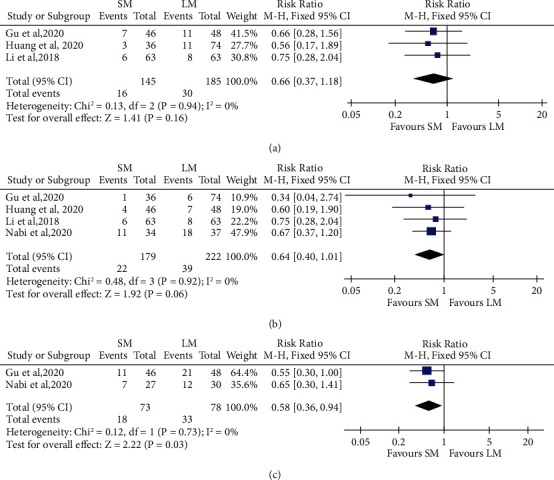
Forest plot presenting the risk ratio of postprocedure GERD measured by symptoms assessment (a), endoscopy (b), and pH monitoring (c) between shorter myotomy and longer myotomy of peroral endoscopic myotomy in achalasia.

Postoperative reflux-related events including symptomatic reflux, reflux esophagitis on endoscopy, and abnormal acid reflux based on pH monitoring were evaluated, respectively. As for the symptomatic reflux, SM showed no significant difference compared with LM (3 studies, *n* = 145 in the SM arm and *n* = 185 in the LM arm, RR 0.66, 95% CI 0.37 to 1.18, *P* = 0.16, Figure 7(a)). Regarding the endoscopic findings, SM showed a lower trend of GERD with borderline significance compared with LM (4 studies, *n* = 179 in the SM arm and *n* = 222 in the LM arm, RR 0.64, 95% CI 0.40 to 1.01, *P* = 0.06, Figure 7(b)). With regard to the abnormal acid reflux based on pH monitoring, SM significantly decreased post-POEM GERD incidence compared with LM (2 studies, *n* = 73 in the SM arm and *n* = 78 in the LM arm, RR 0.58, 95% CI 0.36 to 0.94, *P* = 0.03, Figure 7(c)). As the heterogeneity among studies was low in the above analysis (*I*^2^ = 0%, *P* = 0.94; *I*^2^ = 0%, *P* = 0.92; *I*^2^ = 0%, *P* = 0.73, respectively), we used fixed-effect model for the analysis.

### 3.8. Procedure-Related Adverse Events

We did not carry out meta-analysis in procedure-related adverse events due to insufficient data publication. But we performed a detailed summary of all adverse events, which are available in Table 4. The most common adverse events were insufflation-related events (*n* = 25.3%), such as subcutaneous emphysema, pneumothorax, and pneumoperitoneum. Other common adverse events include bleeding (*n* = 9.3%) and mucosal injury/perforation (*n* = 4.9%). All adverse events were resolved with conservative or endoscopic treatment. No surgery intervention was required and no death was reported.

### 3.9. Reporting Biases and Sensitivity Analysis

Comparing the trial protocols with reported outcomes in the corresponding trial publications and comparing the outcomes reported in the methods and results sections of the trial publications, no reporting bias was found. The sensitivity analysis demonstrated the robustness of all the results by using the leave-one-out method.

### 3.10. Certainty of Evidence

Based on GRADE, the certainty of evidence that the clinical success of SM was noninferior to LM was low due to the limitation of observational studies, which signified that further research was very likely to have an important impact on our confidence in the estimate of effect and was likely to change the estimate. The certainty of evidence that the SM decreased post-POEM GERD incidence compared with LM was moderate due to the nature of RCT and inconsistency of the results, which signified that further research was likely to have an important impact on our confidence in the estimate of effect and may change the estimate. The certainty of evidence that the SM decreased the total procedure time compared with LM was very low due to the limitation of observational studies and significant heterogeneity, which signified that any estimate of effect was very uncertain.

## 4. Discussion

To our knowledge, this is a systematic review and meta-analysis to evaluate the efficacy and safety of SM and compare the clinical outcomes between SM and LM for achalasia. Based on our analysis, we demonstrated that the SM was noninferior to LM in terms of providing clinical success, and it could even lower the incidence of post-POEM GERD regarding to abnormal acid reflux and shorten the total procedure time.

POEM is a novel, minimally invasive, and natural orifice endoscopic technology, involving the process of distal esophagus myotomy via a submucosal tunneling approach [21]. Over the last decade, POEM has prompted a revolutionary shift in achalasia management and has triggered a worldwide dissemination of this new technique [22]. Since its introduction by Inoue et al. [4], LM (approximately 10 cm) has been developed into a common practice on a global scale [13]. But the technological elements of POEM have been continuing to be improved in an attempt to make the procedure safer, more effective, and reproducible [23]. Major technical variations in POEM procedure include myotomy length, anterior versus posterior myotomy approach, and full-thickness versus partial-thickness myotomy [24]. These techniques generally vary with operator expertise and preferences in clinical practice [23]. However, these technical aspects are sometimes affected by patient characteristics. In recent years, studies investigating clinical outcomes in connection with these factors have been increasingly published [8, 11–14]. Although these modifications have technically facilitated the procedure, the effect of esophageal myotomy length on POEM outcomes is still controversial. Therefore, we conduct this systematic review and meta-analysis to evaluate the efficacy and safety of the modified POEM with SM and compare the clinical success rate and incidence of reflux-relative AEs between SM and LM in idiopathic achalasia patients.

At this time, the research has proven that POEM is both effective and safe, with a reported overall clinical success rate more than 90% [25, 26]. Consistent with earlier studies, our results show that POEM treatment produces great symptom alleviation and manometric parameter improvement. Meanwhile, there were no severe AEs in all of the individuals. This study revealed and reaffirmed the fact that the POEM is an effective and safe treatment for those with achalasia.

However, POEM has been especially challenging and time-consuming in complex achalasia such as sigmoid-type esophagus, prior treatments, and presence of submucosal fibrosis. Our current study found that the SM significantly shortened the total procedure time compared with LM. Shorter operating time can potentially reduce the overall expense of the procedure by avoiding the need for additional endoscopic tools. In addition, perioperative AEs, especially gas-related events, have been shown to be fewer in cases with shorter procedure duration [11, 27]. Because SM can make POEM easier than the standard myotomy, it is likely to be a better option for these cases. However, we did not find a significant difference in procedure-related adverse events between SM and LM due to the insufficient data. In addition, the length of hospital stay did not differ significantly between the two groups. Therefore, more studies are needed to demonstrate the benefits of SM in POEM procedure.

As is well known, acquired GERD is a notable deficiency in the development of POEM. Based on the objective measurements, the incidence of post-POEM GERD is reported between 10% and 57%, and it appears to be the main challenge of the operation [28, 29]. Identifying intraprocedural factors that increase the likelihood of the development of post-POEM GERD is conceivably valuable to decrease its incidence. Several studies have confirmed that increased length of gastric myotomy lead to increased incidence of post-POEM GERD [9, 30]. However, a recent meta-analysis found that variations in the myotomy technique do not differ in the incidence of post-POEM GERD and could not recommend modifications to the POEM technique to reduce its rate [31]. In this study, we found that the SM decreased post-POEM GERD incidence compared with LM regarding abnormal acid reflux. It has been proven that the circular muscle may contribute to esophageal shortening due to the spiral-shaped structure and the role that it plays in axial movements [13, 23, 32]. Hence, circular muscle keeps reflux of stomach contents from entering the esophagus and thus pushes refluxate downward and back into the stomach again theoretically. When POEM was conducted with SM, it means that longer circular muscles were remained. As a result, we concluded that this may be the reason why SM can lower the incidence of post-POEM abnormal esophageal acid exposure.

By making use of functional lumen imaging and endoscopic esophageal topography, adjustments and customization to the POEM technique have been made much easier. The increased use of this technology is allowing patients to gain more accurate assessments of the sufficiency of a myotomy by having access to real-time measurements of pressure and compliance of the esophagus. At the present time, there are no set optimal distensibility targets; however, this adjuvant technology will play a significant role in the procedure's future [22].

In this study, there are several limitations. Firstly, it was conducted with only five studies, of which four studies were performed in China, meaning that our results may not applicable universally. Meanwhile, the types of studies included in the meta-analysis were heterogenous with only 2 RCTs and others were observational studies. Secondly, most enrolled individuals are adult type I and type II achalasia meaning that our results may not apply to the type III achalasia. Thirdly, we are unable to compare the long-term efficacy and safety between SM and LM due to the short-term and various follow-up. Therefore, there are several suggestions in future studies. Firstly, large prospective multicenter RCTs with long-term follow-up are needed. Secondly, we recommend that an additional analysis to determine whether there is a difference in the above results between different countries or Asian and Western populations. Thirdly, double scope technique can be utilized to detect the length of esophageal and gastric myotomy to avoid underestimated or overestimated the outcomes [33].

In conclusion, based on our analysis, SM and LM of POEM are comparable in terms of providing treatment efficacy for achalasia patients, whereas less operation time and lower incidence of post-POEM abnormal esophageal acid exposure are observed in SM.

## Figures and Tables

**Table 1:** General characteristics of the studies and patients.

Study	Wang et al., 2015	Li et al., 2018		Huang et al., 2020		Gu et al., 2020		Nabi et al., 2020	
Study design	Prospective cohort	Retrospective cohort		Retrospective cohort		RCT		RCT	
Study period	Jan 2012 to Feb 2013	Jan 2013 to Dec 2016		Jul 2011 to Sep 2017		Feb 2018 to Feb 2019		Jun 2017 to Mar 2019	
Study location	Guangzhou, China	Beijing, China		Shenzhen, China		Changsha, China		Hyderabad, India	
Patients group	SM	SM	LM	SM	LM	SM	LM	SM	LM
Total patients (*n*)	46	63	63	36	74	46	48	34	37
Mean age (years)	36	49.3	45.9	40.8	37.7	43.6	42.8	40.1	41.3
Male, *n* (%)	17 (37)	24 (38)	30 (48)	19 (53)	40 (54)	21 (46)	23 (48)	18 (53)	24 (65)
Symptoms duration (years)	<1 year, 12; 1-5 years, 28; and >5 years, 6	9.4 (0.1-40.0)	9.4 (0.3-30.0)	0.7 (0.2-2.1)	0.7 (0.3-2.5)	5.0 (0.3-34.0)	4.1 (0.3-31.0)	3 (1.5-4.7)	3 (1.0-5.0)
Previous treatments (*n*)	POEM, 1; PD, 7	BTI or PD or HM, 23	BTI or PD or HM, 18	BTI, 2; PD, 7	BTI, 3; PD, 9	None	None	PD, 12	PD, 9
Chicago classification, type I/type II/type III	26/19/1	16/45/2	9/52/2	12/24/0	26/48/0	0/46/0	0/48/0	12/22/0	13/24/0
Follow-up (months)	3	20.1 (6-48)	23.6 (6-48)	26.8 (8-54.3)	29.5 (6-58.8)	12	12	12	12

Continuous variables presented as mean (SD) or Median (IQR). SM: short myotomy; LM: long myotomy; POEM: peroral endoscopic myotomy; PD: pneumatic dilatation; BTI: botulinum toxin injection; SD: standard deviation; IQR: interquartile range.

**Table 2:** Details of the POEM procedures.

Study	Wang et al., 2015	Li et al., 2018		Huang et al., 2020		Gu et al., 2020		Nabi et al., 2020	
Group	SM	SM	LM	SM	LM	SM	LM	SM	LM
Total patients (n)	46	63	63	36	74	46	48	34	37
Procedure time (minutes)	52 (30-120)	39.5 (21-74)	49.2 (23-120)	46.6 ± 18.5	62.1 ± 25.2	31.2 ± 15.3	45.6 ± 16.2	44.03 ± 13.78	72.43 ± 27.28
Myotomy direction, A/P	NR	NR	NR	NR	NR	0/46	0/48	34/0	37/0
Tunnel length (cm)	6.8 (4.0-10.0)	7.6 (6-8)	11.8 (10-14)	8.6 ± 1.3	15.1 ± 2.9	NR	NR	NR	NR
Myotomy length (cm)	E: 4.3 (3.0-5.5); G: 1.1 (1.0-2.0); and T: 5.4 (3.5-7.5)	E: 2.9 (2-4); G: 2.0 (1-3); and T: 4.8 (3-6)	E: 6.9 (5-9); G: 2.3 (2-4); and T: 9.2 (8-11)	E: 4.0 ± 0.7; G: 2.1 ± 0.3; and T: 6.0 ± 0.6	E: 8.2 ± 2.7; G: 3.2 ± 1.2; and T: 11.5 ± 3.1	T: 5.66 ± 0.14	T: 10.1 ± 0.54	E: 2.76 ± 0.41; G: 2.70 ± 0.73	E: 7.97 ± 2.40; G: 2.84 ± 0.63
Myotomy extent	SCM, 15; FTM, 31	PFTM, 56; SCM, 1; and FTM, 6	PFTM, 50; SCM, 5; and FTM, 8	NR	NR	SCM, 46	SCM, 48	FTM, 34	SCM and FTM, 37
Hospitalization (days)	2.9 (2-6)	NR	NR	9.9 ± 2.4	9.3 ± 2.9	7.0 ± 0.9	6.5 ± 1.6	2.82 ± 0.67	2.81 ± 0.70

Continuous variables presented as mean ± SD or median (IQR). SM: short myotomy; LM: long myotomy; NR: not reported; E: esophageal; G: gastric; T: total; A/P: anterior/posterior; SCM: selective circular myotomy; FTM: full thickness myotomy; PFTM: progressive full thickness myotomy; SD: standard deviation; IQR: interquartile range.

**Table 3:** Outcomes reported in the included studies.

Study	Wang et al., 2015	Li et al., 2018		Huang et al., 2020		Gu et al., 2020		Nabi et al., 2020	
Group	SM	SM	LM	SM	LM	SM	LM	SM	LM
Total patients (*n*)	46	63	63	36	74	46	48	34	37
Technical success, *n* (%)	46 (100)	63 (100)	63 (100)	36 (100)	74 (100)	46 (100)	48 (100)	34 (100)	37 (100)
Clinical success, *n*/*N* (%)	46/46 (100)	56/57 (98.2)	55/56 (98.2)	34/36 (94.4)	68/74 (91.9)	44/46 (95.7)	45/48 (93.8)	32/33 (97.0)	29/31 (93.5)
Pre-Eckardt score	8.4 ± 3.2	7.9 (5-11)	7.3 (4-11)	7.1 ± 1.6	7.5 ± 1.9	7.56 ± 1.56	7.12 ± 1.68	6.02 ± 1.33	6.75 ± 1.32
Post-Eckardt score	2.7 ± 1.9	1.1 (0-4)	1.0 (0-4)	1.3 ± 1.2	1.6 ± 1.3	0.76 ± 0.51	0.72 ± 0.42	0.935 ± 0.929	0.818 ± 0.983
Pre-LESP (mm Hg)	39.4 ± 10.1	27.8 (0.7-57.7)	29.6 (9.6-50.4)	41.8 ± 14.3	39.7 ± 13.9	33.5 ± 5.0	32.4 ± 5.3	NR	NR
Post-LESP (mm Hg)	24.4 ± 9.1	15.6 (1.5 -35.7)	17.7 (3.0-38.8)	15.9 ± 3.2	13.3 ± 5.7	11.8 ± 4.4	12.1 ± 3.9	NR	NR
Pre-IRP (mm Hg)	38.6 ± 10.4	NR	NR	NR	NR	23.2 ± 4.8	21.5 ± 4.6	26.40 ± 13.93	28.50 ± 11.01
Post-IRP (mm Hg)	25.7 ± 9.6	NR	NR	NR	NR	10.1 ± 2.4	9.7 ± 2.6	8.60 ± 1.30	7.44 ± 4.30
Pre-DBC (cm)	4.9 ± 2.2	NR	NR	7.91 ± 2.64	8.16 ± 4.37	5.9 ± 1.0	5.6 ± 0.8	NR	NR
Post-DBC (cm)	4.1 ± 2.3	NR	NR	NR	NR	3.7 ± 0.5	3.5 ± 0.5	NR	NR
Pre-HBC (cm)	5.4 ± 2.1	NR	NR	NR	NR	NR	NR	12.99 ± 5.40	11.21 ± 5.36
Post-HBC (cm)	2.6 ± 1.8	NR	NR	NR	NR	NR	NR	1.90 ± 2.39	2.31 ± 1.71

Continuous variables presented as mean ± SD or median (IQR). SM: short myotomy; LM: long myotomy; LESP: lower esophageal sphincter pressure; IRP: integrated relax pressure; DBC: diameter of barium column; HBC: height of barium column; SD: standard deviation; IQR: interquartile range.

**Table 4:** Detailed procedure-related adverse events and reflux adverse events.

Study	Group	Total patients (*n*)	Perioperative adverse events, *n* (%)	Postprocedure GERD, *n* (%)
Wang et al., 2015	SM	46	Bleeding, 7 (15.2); perforation, 6 (13.0); pneumothorax, 14 (30.4); pneumoperitoneum, 12 (26.1); and emphysema, 17 (37.0)	Symptoms or endoscopy, 7 (15.2)
Li et al., 2018	SM	63	Mucosal injury, 4 (6.3); pneumoperitoneum, 2 (3.2); and fever (temperature > 38.0°C), 6 (9.5)	Symptoms, 6 (9.5); endoscopy, 6 (9.5)
	LM	63	Mucosal injury, 5 (7.9); pneumothorax, 1 (1.6); pneumoperitoneum, 3 (4.8); pneumomediastinum, 1 (1.6); subcutaneous emphysema, 24 (38.1); and fever (temperature > 38.0°C), 7 (11.1)	Symptoms, 8 (12.7); endoscopy, 8 (12.7)
Huang et al., 2020	SM	36	Major bleeding, 2 (5.6); pneumothorax, 1 (2.8)	Symptoms, 3 (8.3); endoscopy, 1 (2.8)
	LM	74	Major bleeding, 3 (4.1); pneumothorax, 2 (2.7); and mucosal perforation, 1 (1.4)	Symptoms, 11 (14.9); endoscopy, 6 (8.1)
Gu et al., 2020	SM	46	None	Symptoms, 7 (15.2); endoscopy, 4 (8.7); and pH, 11 (23.9)
	LM	48	Mucosal injuries, 1 (2.08)	Symptoms, 11 (22.9); endoscopy, 7 (14.6); and pH, 21 (43.8)
Nabi et al., 2020	SM	34	Subcutaneous emphysema, 4 (11.76); capnoperitoneum requiring decompression, 3 (8.82); retroperitoneal CO2, 4 (11.76); minor bleeding episodes, 12 (35.29); and mucosal injuries requiring clipping, 1 (2.94)	Endoscopy, 11 (32.4); pH, 7 (25.92)
	LM	37	Subcutaneous emphysema, 4 (11.76); capnoperitoneum requiring decompression, 3 (8.10); retroperitoneal CO 2, 2 (5.40); minor bleeding episodes, 17 (45.94); mucosal injuries requiring clipping, 1 (2.70)	Endoscopy, 18 (48.6); pH, 12 (40.00)

SM: short myotomy; LM: long myotomy.

## Data Availability

The data that support the results of this study are available from Dr. Han Zhang (443191590@qq.com) upon reasonable request.

## Supplementary Materials

Supplementary Materials Supplementary tables including electronic database search strategy, reasons for the excluded articles, and quality assessment of included studies are available online.

## References

[1] Vaezi M. F., Pandolfino J. E., Yadlapati R. H., Greer K. B., Kavitt R. T. (2020). ACG clinical guidelines: diagnosis and management of achalasia. *The American Journal of Gastroenterology*.

[2] Boeckxstaens G. E., Zaninotto G., Richter J. E. (2014). Achalasia. *Lancet*.

[3] Zaninotto G., Bennett C., Boeckxstaens G. (2018). The 2018 ISDE achalasia guidelines. *Diseases of the Esophagus*.

[4] Inoue H., Minami H., Kobayashi Y. (2010). Peroral endoscopic myotomy (POEM) for esophageal achalasia. *Endoscopy*.

[5] Barbieri L. A., Hassan C., Rosati R., Romario U. F., Correale L., Repici A. (2015). Systematic review and meta-analysis: efficacy and safety of POEM for achalasia. *United European Gastroenterology Journal*.

[6] Talukdar R., Inoue H., Nageshwar Reddy D. (2015). Efficacy of peroral endoscopic myotomy (POEM) in the treatment of achalasia: a systematic review and meta-analysis. *Surgical Endoscopy*.

[7] Stavropoulos S. N., Desilets D. J., Fuchs K. H. (2014). Per-oral endoscopic myotomy white paper summary. *Gastrointestinal Endoscopy*.

[8] Wang J., Tan N., Xiao Y. (2015). Safety and efficacy of the modified peroral endoscopic myotomy with shorter myotomy for achalasia patients: a prospective study. *Diseases of the Esophagus*.

[9] Familiari P., Greco S., Gigante G. (2016). Gastroesophageal reflux disease after peroral endoscopic myotomy: analysis of clinical, procedural and functional factors, associated with gastroesophageal reflux disease and esophagitis. *Digestive Endoscopy*.

[10] Grimes K. L., Bechara R., Shimamura Y., Ikeda H., Inoue H. (2020). Gastric myotomy length affects severity but not rate of post-procedure reflux: 3-year follow-up of a prospective randomized controlled trial of double-scope per-oral endoscopic myotomy (POEM) for esophageal achalasia. *Surgical Endoscopy*.

[11] Li L., Chai N., Linghu E. (2019). Safety and efficacy of using a short tunnel versus a standard tunnel for peroral endoscopic myotomy for Ling type IIc and III achalasia: a retrospective study. *Surgical Endoscopy*.

[12] Huang S., Ren Y., Peng W. (2020). Peroral endoscopic shorter versus longer myotomy for the treatment of achalasia: a comparative retrospective study. *Esophagus*.

[13] Gu L., Ouyang Z., Lv L., Liang C., Zhu H., Liu D. (2021). Safety and efficacy of peroral endoscopic myotomy with standard myotomy versus short myotomy for treatment-naïve patients with type II achalasia: a prospective randomized trial. *Gastrointestinal Endoscopy*.

[14] Nabi Z., Ramchandani M., Sayyed M. (2021). Comparison of short versus long esophageal myotomy in cases with idiopathic achalasia: a randomized controlled trial. *Journal of Neurogastroenterology and Motility*.

[15] Page M. J., McKenzie J. E., Bossuyt P. M. (2021). The PRISMA 2020 statement: an updated guideline for reporting systematic reviews. *International Journal of Surgery*.

[16] Eckardt V. F., Aignherr C., Bernhard G. (1992). Predictors of outcome in patients with achalasia treated by pneumatic dilation. *Gastroenterology*.

[17] Stang A. (2010). Critical evaluation of the Newcastle-Ottawa scale for the assessment of the quality of nonrandomized studies in meta-analyses. *European Journal of Epidemiology*.

[18] Higgins J. P., Altman D. G., Gøtzsche P. C. (2011). The Cochrane Collaboration's tool for assessing risk of bias in randomised trials. *BMJ*.

[19] Guyatt G. H., Oxman A. D., Vist G. E. (2008). GRADE: an emerging consensus on rating quality of evidence and strength of recommendations. *BMJ*.

[20] Hozo S. P., Djulbegovic B., Hozo I. (2005). Estimating the mean and variance from the median, range, and the size of a sample. *BMC Medical Research Methodology*.

[21] Mittal C., Wagh M. S. (2017). Technical advances in per-oral endoscopic myotomy (POEM). *The American Journal of Gastroenterology*.

[22] Haisley K. R., Swanström L. L. (2021). The modern age of POEM: the past, present and future of per-oral endoscopic myotomy. *Journal of Gastrointestinal Surgery*.

[23] Dai Q., Korimilli A., Thangada V. K. (2006). Muscle shortening along the normal esophagus during swallowing. *Digestive Diseases and Sciences*.

[24] Jonica E. R., Wagh M. S. (2021). Length of esophageal myotomy during peroral endoscopic myotomy for achalasia: it's okay to take the shortcut. *Gastrointestinal Endoscopy*.

[25] Crespin O. M., Liu L., Parmar A. (2017). Safety and efficacy of POEM for treatment of achalasia: a systematic review of the literature. *Surgical Endoscopy*.

[26] Evensen H., Kristensen V., Larssen L., Sandstad O., Hauge T., Medhus A. W. (2019). Outcome of peroral endoscopic myotomy (POEM) in treatment-naive patients. A systematic review. *Scandinavian Journal of Gastroenterology*.

[27] Haito-Chavez Y., Inoue H., Beard K. W. (2017). Comprehensive analysis of adverse events associated with per oral endoscopic myotomy in 1826 patients: an international multicenter study. *The American Journal of Gastroenterology*.

[28] Sanaka M. R., Thota P. N., Parikh M. P. (2019). Peroral endoscopic myotomy leads to higher rates of abnormal esophageal acid exposure than laparoscopic Heller myotomy in achalasia. *Surgical Endoscopy*.

[29] Repici A., Fuccio L., Maselli R. (2018). GERD after per-oral endoscopic myotomy as compared with Heller's myotomy with fundoplication: a systematic review with meta-analysis. *Gastrointestinal Endoscopy*.

[30] Inoue H., Shiwaku H., Kobayashi Y. (2020). Statement for gastroesophageal reflux disease after peroral endoscopic myotomy from an international multicenter experience. *Esophagus*.

[31] Mota R., de Moura E., de Moura D. (2021). Risk factors for gastroesophageal reflux after POEM for achalasia: a systematic review and meta-analysis. *Surgical Endoscopy*.

[32] Patel N., Jiang Y., Mittal R. K., Kim T. H., Ledgerwood M., Bhargava V. (2015). Circular and longitudinal muscles shortening indicates sliding patterns during peristalsis and transient lower esophageal sphincter relaxation. *American Journal of Physiology*.

[33] Khashab M. A., Kumbhari V., Azola A. (2015). Intraoperative determination of the adequacy of myotomy length during peroral endoscopic myotomy (POEM): the double-endoscope transillumination for extent confirmation technique (DETECT). *Endoscopy*.

