# Supplementary material for: Safety and Efficacy of Peroral Endoscopic Shorter Myotomy versus Longer Myotomy for Patients with Achalasia: A Systematic Review and Meta-analysis
Source: Gastroenterol Res Pract. 2022 Mar 30;2022:6770864. doi: 10.1155/2022/6770864 (PMC8986442; doi:10.1155/2022/6770864)
Supplement: Supplementary Materials — Supplementary tables including electronic database search strategy, reasons for the excluded articles, and quality assessment of included studies are available online. [file 6770864.f1.docx]

Supplementary Table 1. Electronic database search strategy

| Database | Search query | Records identified |
| --- | --- | --- |
| PubMed | (((((poem) OR (peroral endoscopic myotomy)) OR (per-oral endoscopic myotomy)) OR (per oral endoscopic myotomy)) AND (achalasia)) [All Fields] | 930 |
| EMBASE | ('esophagus achalasia'/exp OR achalasia:ab,ti) AND (poem:ab,ti OR 'peroral endoscopic myotomy':ab,ti OR 'per-oral endoscopic myotomy':ab,ti OR 'per oral endoscopic myotomy':ab,ti) | 2048 |
| Cochrane Library | ("achalasia" AND ("poem" OR "peroral endoscopic myotomy" OR "per-oral endoscopic myotomy" OR "per oral endoscopic myotomy") ) [Title Abstract Keyword] | 138 |
| Web of Science  Core Collection | (TOPIC: (poem) OR TOPIC: (peroral endoscopic myotomy) OR TOPIC: (per-oral endoscopic myotomy) OR TOPIC: (per oral endoscopic myotomy) ) AND TOPIC: (achalasia)  Timespan: All years. Indexes: SCI-EXPANDED, SSCI. | 1138 |

Date of search: May 28, 2021

Supplementary Table 2: The reasons for the excluded articles after full text review.

| Reason for exclusion |
| --- |
| Protocol for an RCT (n = 8) |
| Incorrect intervention or comparator (n = 2) |
| Duplicate data from the same center/author (n = 6) |
| Incorrect definition of clinical success (n = 1) |
| Editorial, letter to editor, or expert comment (n = 5) |
| Conference abstract without available full text (n = 5) |

Supplementary Table 3. Quality assessment of the non-randomized studies by the Newcastle-Ottawa Scale (NOS)

| Study | Newcastle-Ottawa Scale items | | | | | | | | Overall stars |
| --- | --- | --- | --- | --- | --- | --- | --- | --- | --- |
|  | Selection | | | | Comparability | Outcome | |  |  |
|  | 1 | 2 | 3 | 4 | 5 | 6 | 7 | 8 |  |
| Wang et al, 2015 | ✭ |  | ✭ | ✭ |  | ✭ | ✭ | ✭ | 6 |
| Li et al, 2018 | ✭ | ✭ | ✭ | ✭ |  | ✭ | ✭ | ✭ | 7 |
| Huang et al,2020 | ✭ | ✭ | ✭ | ✭ |  | ✭ | ✭ | ✭ | 7 |

– A study can be awarded a maximum of one star for each numbered item within the Selection and outcome categories.

– A maximum of two stars can be given for Comparability.

Supplementary Table 4. Quality assessment of the randomized studies by the Cochrane Collaboration's tool

| Study | Cochrane Collaboration's tool items | | | | | | | Overall |
| --- | --- | --- | --- | --- | --- | --- | --- | --- |
|  | 1 | 2 | 3 | 4 | 5 | 6 | 7 |  |
| Wang et al, 2015 | Low | Low | Low | Low | Low | Low | Unclear | Low |
| Li et al, 2018 | Low | Low | Low | Low | Low | Low | Unclear | Low |
